# Preparation and Characterization of Polylactic Acid/Nano Hydroxyapatite/Nano Hydroxyapatite/Human Acellular Amniotic Membrane (PLA/nHAp/HAAM) Hybrid Scaffold for Bone Tissue Defect Repair

**DOI:** 10.3390/ma16051937

**Published:** 2023-02-26

**Authors:** Zhilin Jia, Hailin Ma, Jiaqi Liu, Xinyu Yan, Tianqing Liu, Yuen Yee Cheng, Xiangqin Li, Shuo Wu, Jingying Zhang, Kedong Song

**Affiliations:** 1 State Key Laboratory of Fine Chemicals, Dalian R&D Center for Stem Cell and Tissue Engineering, Dalian University of Technology, Dalian 116024, China; 2 Department of Hematology, The First Affiliated Hospital of Dalian Medical University, Dalian 116011, China; 3 Institute for Biomedical Materials and Devices, Faculty of Science, University of Technology Sydney, Sydney, NSW 2007, Australia; 4 Department of Medical Oncology, Cancer Hospital of Dalian University of Technology, Liaoning Cancer Hospital & Institute, Shenyang 110042, China; 5 Key Laboratory of 3D Printing Technology in Stomatology, The First Dongguan Affiliated Hospital, Guangdong Medical University, Dongguan 523808, China

**Keywords:** human acellular amniotic membrane, acellular process, osteoblasts, polylactic acid, hydroxyapatite

## Abstract

Bone tissue engineering is a novel and efficient repair method for bone tissue defects, and the key step of the bone tissue engineering repair strategy is to prepare non-toxic, metabolizable, biocompatible, bone-induced tissue engineering scaffolds of suitable mechanical strength. Human acellular amniotic membrane (HAAM) is mainly composed of collagen and mucopolysaccharide; it has a natural three-dimensional structure and no immunogenicity. In this study, a polylactic acid (PLA)/Hydroxyapatite (nHAp)/Human acellular amniotic membrane (HAAM) composite scaffold was prepared and the porosity, water absorption and elastic modulus of the composite scaffold were characterized. After that, the cell–scaffold composite was constructed using newborn Sprague Dawley (SD) rat osteoblasts to characterize the biological properties of the composite. In conclusion, the scaffolds have a composite structure of large and small holes with a large pore diameter of 200 μm and a small pore diameter of 30 μm. After adding HAAM, the contact angle of the composite decreases to 38.7°, and the water absorption reaches 249.7%. The addition of nHAp can improve the scaffold’s mechanical strength. The degradation rate of the PLA+nHAp+HAAM group was the highest, reaching 39.48% after 12 weeks. Fluorescence staining showed that the cells were evenly distributed and had good activity on the composite scaffold; the PLA+nHAp+HAAM scaffold has the highest cell viability. The adhesion rate to HAAM was the highest, and the addition of nHAp and HAAM could promote the rapid adhesion of cells to scaffolds. The addition of HAAM and nHAp can significantly promote the secretion of ALP. Therefore, the PLA/nHAp/HAAM composite scaffold can support the adhesion, proliferation and differentiation of osteoblasts in vitro which provide sufficient space for cell proliferation, and is suitable for the formation and development of solid bone tissue.

## 1. Introduction

Bone tissue engineering involves developing a scaffold that can simulate the microenvironment of bone tissue by combining the principles of engineering and bone science, and compound seed cells with a scaffold [1] so as to further use the physiological reactions of the body to repair tissue damage. Ideal bone tissue engineering scaffolds can provide good mechanical strength to withstand stress load and a suitable porous structure for bone cell migration and differentiation; they can also promote bone conduction and strengthen bone integration with receptors [2].

In the study of bone tissue engineering, biocompatible scaffolds can provide structural guidance and necessary stress load for the repair of large area bone defects. Therefore, selecting an appropriate scaffold material is a key step in the strategy of bone tissue construction [3]. The emergence and application of polylactic acid (PLA) provides a new direction for realizing this goal; it is effective not only due to its good biocompatibility and degradation [4], but also because the three-dimensional regularity of PLA can be controlled in the polymerization process, thus indirectly controlling the physical and chemical properties of the material. Controlling these properties, such as mechanical properties, thermal stability and degradation characteristics [5], means that PLA materials can be “customized” to meet certain requirements through the control of the PLA synthesis process. Hydroxyapatite (nHAp) is a kind of inorganic material widely used in tissue engineering. Its chemical composition and crystal structure are similar to inorganic components in human bones, and it is also the main organic component of natural bones [6]. The nHAp has superior biological activity and biocompatibility, as well as good bone conductivity, which can well support the generation of new bone [7]. The addition of nHAp can also significantly improve the mechanical properties of scaffolds and induce cell differentiation into osteoblasts [8].

Amniotic membrane (AM) is a colorless and transparent membrane free of blood vessels, nerves and lymphatics obtained from the innermost layer of the placenta [9]. It is characterized by low immune repulsion, low inflammatory response and inhibition of leukocyte penetration. It is widely used in the repair of eye trauma [10] and skin trauma [11]. Human acellular amniotic membrane (HAAM) is obtained after removal of fresh amniotic epithelial cells. It preserves the basal layer and stromal layer and is rich in collagen and mucopolysaccharide [12]. Acellular amniotic membrane is considered a potential biomaterial which can be used directly in tissue repair or as a substrate for autologous/allograft [13]. As a tissue engineering material, it has the following three advantages: (1) low immune repulsion and low inflammatory response [14,15]; (2) natural three-dimensional physical structure; and (3) promotion of cell adhesion [16]. Most collagen in acellular amniotic membrane exists in the form of collagen fibers which are crisscrossed to form a collagen network with a natural three-dimensional structure [17]. The diameter of the pores in the collagen network ranges from several microns to tens of microns. It provides more abundant cell adhesion sites [18], which is conducive to the adhesion and spreading of cells on the acellular amniotic membrane [19]. On the other hand, it is also conducive to the timely elimination of cell metabolites and the smooth transfer of nutrients, providing a microenvironment for the balance of supply and demand. The basal layer of acellular amniotic membrane consists of type I collagen, type III collagen and glycoprotein secreted by amniotic epithelial cells [20]. Collagen maintains the mechanical properties of bone, supports the metabolism of bone, plays an important role in the maturation and development of bone tissue [21], and can regulate the osteogenic differentiation of cells. Human dental papilla cells inoculated into acellular amniotic membrane showed higher ALP activity [22]. 

In fact, one of the most noteworthy advantages of seed-cell based bone tissue engineering is the diversification of seed cell sources in the process of new bone reconstruction independent of the quantity and quality of autologous bone progenitor cells [23], which has an unusual significance for a patient population with reduced bone progenitor cell reserves. Osteoblasts (OBs) are derived from bone progenitor cells that are widespread in bone marrow and periosteum. Osteoblasts perform the function of generating bone tissue by synthesizing, secreting, arranging and mineralizing the extracellular matrix [24]. Osteoblasts can secrete a large amount of organic extracellular matrix, mainly type I collagen and type II collagen [25]. Osteoblasts also secrete a variety of enzymes, cytokines, and glycoproteins in alkaline phosphatase (ALP) and gene expression markers, including alkaline phosphatase (ALP), osterix, col1a1, and alkaline phosphatase [26]. They also secrete bone salivary protein and macrophage colony-stimulating factor. The use of osteoblasts during tissue-engineered bone construction has been shown to improve the rate and quality of bone tissue regeneration [27].

In this study, acellular amniotic membrane was used to prepare tissue engineering scaffolds with high nutrient transport efficiency, high biocompatibility and osteogenic ability. Firstly, acellular amniotic membrane was prepared by four different acellular methods, and then the physicochemical properties and biocompatibility of acellular amniotic membrane were characterized. Secondly, PLA/nHAp/HAAM composite scaffold was prepared, and the porosity and protein adsorption capacity of the composite scaffold were characterized. Finally, osteoblasts were used to construct the cell–scaffold complex, and the bioactivity and osteogenic ability of the complex were characterized, aiming to use the composite scaffold as a favorable material for bone defect repair.

## 2. Materials and Methods

### 2.1. Materials

Fresh human amniotic membrane was obtained from the Second Hospital of Dalian Medical University, with the knowledge and consent of the woman donator aged between 25 and 35 years old. The SPF Animal Experiment Center of Dalian Medical University was used to provide newborn SD rats. All biological experiments have been approved by the Biological and Medical Ethics Committee of Dalian University of Technology (Approval No.: DUTSCE230210-01). The reagents used for decellularization, calcein and Hoechst were purchased from Sigma-Aldrich Inc. (St. Louis, MO, USA). The reagents used for cell culture were purchased from Gibco, USA. Polylactic acid (PLA) was purchased from Jinan Dagang Bioengineering Co., Ltd. (Jinan, China), and nanoscale hydroxyapatite (nHAp) was purchased from Aladdin. Hematoxylin-eosin dye solution, a diquinoline formic acid assay kit and an alkaline phosphatase kit were purchased from Shanghai Beyotime Biotechnology Co., Ltd. (Shanghai, China) Alizarin red was purchased from Beijing Regen Biotechnology Co., Ltd. (Beijing, China).

### 2.2. Preparation and Characterization of the HAAM by Various Methods

In this experiment, four methods were used to decellularize the human amniotic membrane. (1) 1% TritonX-100 solution +0.25% trypsin/EDTA solution: Immerse fresh amniotic membrane in 1% TritonX-100 solution and place in a gas bath incubator for constant temperature oscillation for 36 h. After removal, rinse with PBS solution, then add 0.25% trypsin/EDTA solution and oscillate at 37 °C for 4 h. Remove and rinse with PBS solution [28]. (2) 5% TritonX-100 solution +0.25% trypsin/EDTA solution: As above, replace 1% TritonX-100 solution with 5% TritonX-100 solution, and reduce the oscillation time to 24 h after adding 5% TritonX-100 solution. (3) 0.5 mol/L sodium hydroxide scraping: Evenly spread the epithelial side of the PBS-rinsed amniotic membrane in the center of the Petri dish, and use a cotton swab soaked with 0.5 mol/L NaOH to gently and evenly scrape the surface of the amniotic membrane for 2 min. Immediately after scraping, wash the lye and cell debris with a large amount of PBS solution, and wash twice for 10 min each time [29]. (4) 3 mol/L hypertonic saline +0.25% trypsin/EDTA solution: Immerse the PBS-rinsed amniotic membrane in 3 mol/L NaCl solution and then place in a gas bath incubator for constant temperature oscillation for 30 min. After removal, rinse with PBS solution, then add 0.25% trypsin digestion solution and shake at 37 °C for 4 h. Remove and rinse with PBS solution [30].

#### 2.2.1. Scanning Electron Microscopy (SEM) 

The morphology of the human acellular amniotic membrane (HAAM) was characterized by a scanning electron microscope (QUANTA450, FEI, Hillsboro, OR, USA). The decellularized human amniotic membrane was cut into 1 cm^3^, washed three times with PBS, and soaked in 2.5% glutaraldehyde solution for fixation. Gradient dehydration with alcohol (30%, 50%, 70%, 90%, 100%) was used, and the dehydration time of each stage was 30 min; the membrane was fixed on the stage and purged with nitrogen to remove surface debris. Then, the HAAM was sprayed with a thin gold layer and photographed to collect images at different magnifications. The morphology and pore size of the scaffolds were analyzed from the SEM images.

#### 2.2.2. Hematoxylin and Eeosin (H&E) Staining Assay

The decellularized human amniotic membrane was placed in a culture dish, fixed with 4% paraformaldehyde for 20 min and rinsed with purified water twice. Hematoxylin dye solution was added for 10 min, excess dye solution was eluted with purified water, and the membrane was rinsed with 95% ethanol solution three times. It was then rinsed with purified water twice, eosin dye solution was added for 2 min, then the membrane was rinsed with water and 70% ethanol solution three times and placed under the microscope (OLYMPUS, IX70, Tokyo, Japan) for observation.

#### 2.2.3. Fourier Transform Infrared Spectroscopy (FTIR) Assay

The chemical structure and functional groups of the composite scaffolds were analyzed by Fourier transform infrared spectroscopy (Nicollet 6700, Thermo Fisher, Walthamm, MA, USA). The HAAM was treated with potassium bromide (KBr) for infrared detection, and infrared spectra in the range of 4500 to 500 cm^−1^ were obtained.

#### 2.2.4. Water Absorbency Detection

The four groups of HAAM were cut into similar sizes and dried. The initial weight of acellular amniotic membranes in each group was weighed by an electronic balance and recorded as m_0_. Then, the HAAMs in each group were placed in a Petri dish and ddH_2_O was added. The acellular amniotic membranes were extracted with tweezers at different times, water was wiped off with absorbent paper, and the weight was recorded as m_t_. The following formula is used to calculate water absorption.

(1) $$A=\frac{m_{t}{-\;m}_{0}}{m_{0}}\times 100\%$$

#### 2.2.5. Biocompatibility Assay

PC12 cells were inoculated on the HAAM with a density of 5 × 10^5^ cells/mL, and the viability of cells was determined by the Methylthiazolyldiphenyl-tetrazolium (MTT) method. The distribution and morphology of cells were observed by calcein fluorescence staining.

#### 2.2.6. Processing of the HAAM

The acellular amniotic membranes were laid in culture dishes, pre-frozen in a −20 °C refrigerator for 24 h, and freeze-dried for 36 h. The freeze-dried acellular amniotic membranes were cut into pieces with surgical scissors and transferred to a mortar for grinding. The HAAM powder was screened (120 mesh), dried and stored for later use.

### 2.3. Preparation and Characterization of the Composite Scaffolds

In this experiment, the composite scaffold was prepared by non-solvent-induced phase separation. Methylene chloride solution with a certain concentration of PLA was prepared in a water bath at 50 °C until the PLA was completely dissolved in methylene chloride until it was clarified. In this paper, n-hexane was added by drip addition, PLA solution was quickly stirred on a magnetic agitator, and n-hexane equal to dichloromethane was slowly added by drip with a needle. After that, a certain amount of nHAp was added to the solution, which was rapidly stirred in a magnetic agitator for premixing and then further mixed in an ultrasonic oscillator until the nHAp was evenly dispersed in the solution. The prepared mixed solution was poured into a glass mold and placed in a −20 °C refrigerator for phase separation for 24 h. After phase separation, the dilute phase was abandoned, the concentrated phase colloid was removed by breaking the mold, and the solvent was extracted clean by placing the extracted material in anhydrous methanol for 24 h. The extracted material was dried in a vacuum drying oven for 24 h to obtain dry PLA/nHAp composites. The preparation of PLA/nHAp/HAAM is basically same as the above steps; the only difference is that after adding nHAp, a certain amount of freeze-dried HAAM powder is added into the solution to mix and separate the phase.

#### 2.3.1. Scanning Electron Microscopy (SEM)

The morphology of the PLA/nHAp/HAAM scaffolds was characterized by a scanning electron microscope (QUANTA450, FEI, USA). The hybrid scaffolds were cut into thin slices of 1 mm × 1 mm. They were fixed on the stage by conductive tape, purged with nitrogen to remove surface debris and sprayed with gold by a vacuum. The scaffold morphology and pore size were observed under a tungsten lamp scanning electron microscope (SEM magnification ranges from 40× to 1000×).

#### 2.3.2. Orthogonal Experiment of Protein Adsorption

The protein adsorption capacity of the composite scaffold was determined to characterize the biocompatibility. The PLA concentration, nHAp concentration and HAAM concentration were selected as three factors of the orthogonal experiment, and each factor had three levels. The following table (Table 1) lists the specific parameters:

Bicinchoninic acid (BCA) working solution was prepared, an appropriate amount of BCA working solution was prepared according to the instructions, and the standard curve of protein was drawn. The scaffolds of a certain weight were weighed and denoted as m, and 3 mL of solution containing 0.2 mg/mL BSA was added then gently blown to cause the floating scaffolds to be completely immersed in the liquid. The scaffolds were placed in an incubator at 37 °C for 12 h for the protein adsorption process. The blank group was 0.2 mg/mL BSA solution without scaffolds. After 12 h, 20 μL of sample solution was absorbed, denoted as V, and 200 μL of BCA working solution was added. The BCA working solution stood at 37 °C for 30 min, and the absorbance of each well was measured with an enzyme marker. The protein concentration in the sample solution was obtained by referring to the protein standard curve, which was denoted as ω. The protein adsorption capacity of each scaffold was calculated by the following formula.

$$M_{\mathrm{adsorption}}=\frac{{(\omega }_{\mathrm{blank}}{\;-\;\omega }_{\mathrm{sample}})V}{m_{\mathrm{scaffold}}}$$

The obtained scaffold protein adsorption capacity of each group was put into the orthogonal test table, the influence of each factor on the dependent variable (protein adsorption capacity) was obtained through statistical analysis, and the optimal ratio was determined. 

#### 2.3.3. Contact Angle Testing

The hydrophilic properties of the material were characterized by measuring the contact angle between the material and water through the contact angle tester (OCAH200, DATAPHYSCICS, Filderstadt, Germany). The material was cut into thin slices with a thin blade and further pressed into composite slices; then, the contact angle was measured. The water drop volume was set to 6 μL so that the droplets slowly dropped out to the center of the material. The image was captured quickly, and image analysis software SCA20 was used to obtain the contact angle.

### 2.4. Isolation and Culture of Osteoblasts

Primary osteoblasts were extracted from the cranial cap bones of newborn SD rats at 24 h after birth, and the medium used was low glucose Dulbecco’s modified Eagle’s medium. The separation procedure of osteoblasts was as follows: the cranial cap bones separated from SD rats were cut to less than 1 mm^2^ with surgical shear, and the cranial cap bone fragments were extracted into a centrifuge tube with pipets and centrifuged at 1000 rpm for 5 min, and the superserum was discarded. A volume of 4 mL trypsin solution (0.25%) was added into the centrifuge tube containing the fragments, and the digestion was oscillated and digested at 37 °C for 30 min. Upon completion of digestion, the digestion was terminated by adding twice the volume of medium and centrifuging at 1000 rpm for 5 min; the supernatant was then discarded. Collagenase II solution (1 mg/mL) was added to the centrifuge tube, oscillated and digested for 60 min at 37 °C and centrifuged at 1000 rpm for 5 min; then, the supernatant was discarded. After adding a certain amount of the medium, the bottom cells and fragments of cranial skull were gently blown with pipette to make them evenly dispersed, and the cell suspension was transferred to a culture flask for culture at 37 °C in an incubator with 5% CO_2_.

### 2.5. Detection of Mineralization Ability of Osteoblasts In Vitro

The 4th generation osteoblasts were cultured in 24-well plates for staining observation at the 3rd week, and the hematoxylin-eosin staining method was referred to in Section 2.2.2. (1) For the alizarin red staining observation: Dissolve 1 g Tris in 100 mL ddH_2_O, add a suitable amount of HCl to adjust pH to 8.3, add 0.1 g alizarin red powder and stir until completely dissolved. Discard the old medium in the sample hole, rinse with PBS three times, add 95% ethanol to fix for 10 min, and rinse with ddH_2_O three times. Stain with 2 mL 0.1% alizarin red -Tris-HCl solution for 30 min at 37 °C, wash three times with triple-distilled water, and observe under an optical microscope. (2) The ALP staining observation: Abandon the old medium in the sample hole, wash three times with PBS, join BCIP/NBT dye solution and incubate for 30 min in a 37 °C environment. Wash three times with triple-distilled water, and observe under a microscope.

#### Cell Adhesion Rate

The composite scaffold was cut into 0.5 cm^2^ slices of a thickness of 2 mm, and osteoblasts were inoculated on the scaffold. After being cultured for 4 h in an incubator at 37 °C and 5% CO_2_, the cells were digested and the number of cells was counted by blood cell plates. The cell adhesion rate of materials in each group at 4 h can be calculated by the following formula:

$$\mathrm{Adhesion}\;\mathrm{rate}=\frac{\mathrm{Cell}\;\mathrm{number}\;\mathrm{of}\;\mathrm{digested}}{\mathrm{Cell}\;\mathrm{number}\;\mathrm{of}\;\mathrm{seeded}}\;\times \;100\%$$

### 2.6. Statistical Analysis

Origin 9.0 software was used for statistical data analysis. All experimental data applied to this study were calculated at least three times to decrease experimental errors. All quantitative analysis results are presented as mean ± standard deviation (SD) for n = 3. A Student’s *t*-test was used to assess the differences between the groups. A *p* value < 0.05 was considered statistically significant and a *p* value < 0.01 was considered statistically extremely significant.

## 3. Results and Discussion

### 3.1. Characterization of HAAM

**Figure 1:**
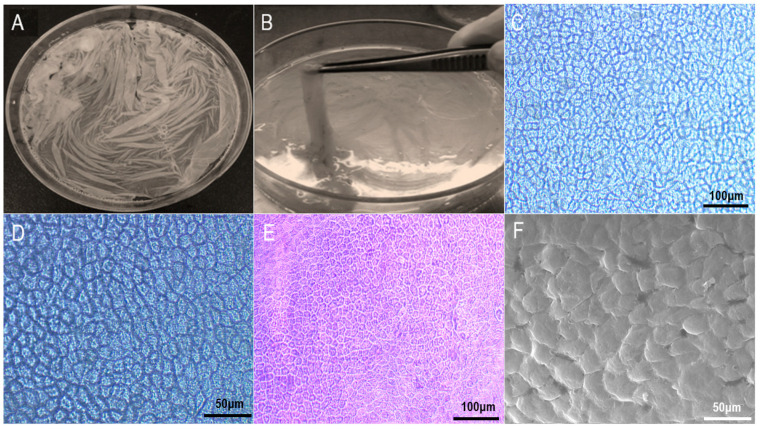
Fresh amniotic membrane macroscopic and microscopic observation. (**A**) Horizontal spread; (**B**) Clamp with tweezers; (**C**) Fresh AM under microscope (×100); (**D**) Fresh AM under microscope (×200); (**E**) HE staining of AM (×100); (**F**) SEM of fresh AM (×2000).

As shown in Figure 1, the fresh amniotic membrane separated from the placenta is a transparent membrane with a density close to that of water, and has strong absorption properties. After absorbing water, it becomes gelatinous. Scanning electron microscopy (Figure 1F) showed that the nuclei of the amniotic epithelial cells were small and evenly distributed in the shape of fish scales. H&E staining (Figure 1E) showed that the amniotic epithelial cells had good light transmission, and the amniotic epithelial cells were evenly distributed and tightly arranged in the shape of paving stones. 

**Figure 2:**
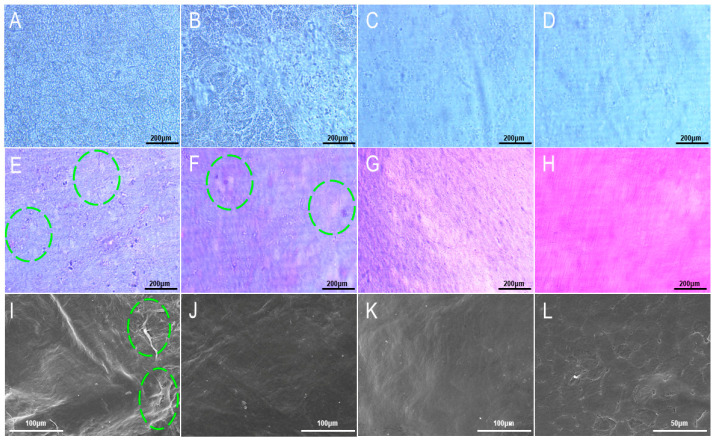
Bright field, HE staining and SEM images of HAAM by different methods. (**A**) Low concentration method after 12 h (×200); (**B**) High concentration method after 12 h (×200); (**C**) Low concentration method complete after 36 h (×200); (**D**) High concentration method complete after 24 h (×200); (**E**) Low concentration TritonX-100 method (×200); (**F**) High concentration TritonX-100 method (×200); (**G**) Hypertonic saline method (×200); (**H**) NaOH scrape method (×200); (**I**) Low concentration TritonX-100 method (×800); (**J**) High concentration TritonX-100 method (×800); (**K**) Hypertonic saline method (×2000); (**L**) NaOH scrape method (×800). The circles represented broken peeling of the amniotic surface.

The treatment time of 1% TritonX-100 combined with 0.25% trypsin/EDTA solution is long, making it easy for the amniotic membrane to deteriorate. Therefore, 5% TritonX-100 was combined with 0.25% trypsin/EDTA solution; with this step, we hoped to shorten the operation time by increasing the concentration of TritonX-100. As shown in Figure 2A,B, after 12 h of treatment, a large area of amniotic epithelial cells in the high concentration group had been shed, while the cells in the low concentration group only appeared loose. This suggests that increasing the concentration of TritonX-100 can indeed accelerate the process of cell removal. In addition, after the complete removal of amniotic epithelial cells from both groups, it was found that increasing the concentration of TritonX-100 did not cause physical damage to the amniotic structure. However, the high concentration method still took 24 h to completely remove the cells, so the high concentration method was still not very efficient.

Although the TritonX-100 method was able to remove epithelial cells more completely under the light microscope, hematoxylin-eosin (H&E) staining showed that the amniotic membrane treated with 1% TritonX-100 still had more dark red epithelial cells. A small number of residual cells were also observed in the amniotic membrane treated with 5% TritonX-100, and the acellular amniotic membrane of the two groups had uneven thickness and some circular perforations (Figure 2E,F). The reason for this may be that the amniotic membranes of these two groups have been treated for a long time, during which the collagen in the amniotic membranes was damaged by toxic TritonX-100, thus leading to perforation and discoloration. As shown in Figure 2G, there were no obvious cell residues on the acellular amniotic membrane treated with hypertonic saline, and the thickness was uniform. The surface structure of the HAAM was very complete, with no obvious traces of damage. Although the NaOH scraping method is to gently scrape the amniotic membrane through cotton swabs, it can be seen in Figure 2H that there are many small scratches on the acellular amniotic membrane, indicating that this physical scraping action still causes damage to the amniotic membrane.

SEM showed that there were many folds on the surface of HAAM in both groups treated with TritonX-100. In addition, Figure 2I,J show that some deformed cells remained on the surface of the acellular amniotic membrane after treatment. As can be seen from Figure 2K, there were no obvious cell residues in the NaOH scraping group, but a large number of turtle cracks and large spalling were generated on the surface, indicating that NaOH scraping had a relatively serious impact on the surface structure of the amniotic membrane. As opposed to the other three groups, the acellular amniotic membrane in the hypertonic saline group had a clean surface; no cells or cell fragments remained, the surface was tight and complete, and no cracks and spallation occurred (Figure 2L). Studies have shown that hypertonic saline solution will not destroy the amniotic membrane itself, but will separate the anchor filaments of cells from the half-desmosomes of epidermal basal cells, thus achieving the effect of cell removal. This indicates that hypertonic saline solution can effectively remove cells while maintaining the integrity of the structure and composition of HAAM.

**Figure 3:**
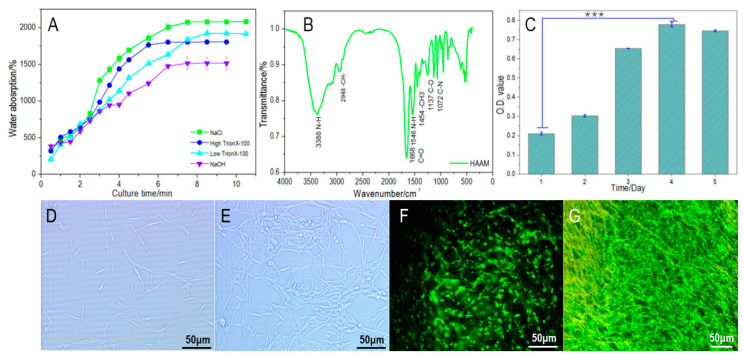
Physicochemical properties and biocompatibility of HAAM. (**A**) Water absorption curve of HAAM by different methods; (**B**) Infrared spectroscopy of HAAM; (**C**) Proliferation activity of PC12 on HAAM, ***: *p* < 0.001; (**D**,**E**) Microscopic observation of PC12 cells cultured 2 and 4 days; (**F**,**G**) The fluorescence staining of PC12 cells cultivated on freezing dried HAAM cultured 2 and 4 days.

A material’s water absorption rate will directly affect its performance in tissue engineering, with high water absorption rates having a higher affinity for the medium; this is conducive to the diffusion of the medium to the scaffold [31] and promotes the interaction of proteins and the provision of more adequate nutrients for the seed cells. As shown in Figure 3A, the water absorption rate of the hypertonic saline group was the highest at 2083%, indicating that the HAAM treated with hypertonic saline could maintain good water absorption performance, and its collagen properties were well preserved. In addition, it can be found in the water absorption curve of the hypertonic saline group that the water absorption rate increases rapidly in the initial stage, indicating that acellular amniotic membrane has a rapid water absorption response when it comes into contact with water. In the process of cell inoculation, the initial period of time is the key stage that determines whether cells can successfully adhere to the material. The water absorption rate of the NaOH scraping group was 1515%, indicating that the NaOH physical scraping method not only caused damage to the surface structure of HAAM but also caused serious damage to the water absorption properties of collagen. The acellular amniotic membrane prepared by the TritonX-100 method in the two groups showed little difference in the final water absorption rate, which was 1804% by the 5% TritonX-100 method and 1921% by the 1% TritonX-100 method. Comparing the two groups, the toxicity of the higher concentration of TritonX-100 was higher than the lower concentration. Therefore, the destruction of the collagen properties of the acellular amniotic membrane was also increased. Therefore, although the high concentration method has an advantage in removal time, it causes greater damage to the performance of the acellular amniotic membrane.

HAAM prepared by the hypertonic saline method will be used for infrared spectroscopy detection. As can be seen from Figure 3B, the absorption peak shown in Table 2 was found in the infrared absorption spectrum of HAAM. The characteristic peaks representing the triple helical structure of collagen are reflected in the infrared spectrum of the acellular amniotic membrane. The characteristic peaks of amide I and amide II bands appear with high intensity, indicating that the secondary structure of collagen is well preserved, the internal hydrogen bond is tightly bound, and the helical structure is not damaged, thereby indicating that the collagen structure is well preserved after acellular treatment.

As shown in Figure 3D, PC12 cells were fully adherent and spread on the second day of culture. By day 4 (Figure 3E), the cells were distributed in sheets that had spread to the bottom of the flask, and a large number of synaptic connections had been established between the cells. In the later stage, acellular amniotic membrane needs to be freeze-dried before being doped into composite scaffolds. Therefore, in order to better verify the performance of HAAM in tissue engineering applications, it should also be freeze-dried before PC12 cells’ seeding. The cell–HAAM complex was treated with Calcein-AM fluorescence staining on day 2 and day 4 after cell seeding. As can be seen from Figure 3F, after 2 days of culture, PC12 cells spread out in a long spindle shape and smoothly distributed regularly on the HAAM. Synapses grew between cells and connected to form a cell network. As shown in Figure 3G, PC12 cells proliferated on day 4 and synapses further developed, forming a complex cell network between cells. These results indicate that human amniotic membrane treated by acellular and freeze-drying still has good cell adhesion and biocompatibility, and cells can adhere, proliferate and maintain high biological activity on it. MTT was used to determine cell proliferation. As can be seen from Figure 3C, cells proliferate slowly in the first 2 days of inoculation, and the absorbance rate reached 0.2083 and 0.3017, respectively. On day 3, cells enter the exponential growth stage and begin to proliferate in large numbers, and the absorbance rate reaches 0.6554. On day 4, the absorbance reached 0.7775, 3.7 times higher than that on the first day of inoculation, and the number of cells increased significantly and reached their maximum. The absorbance rate of cells decreased slightly to 0.7473 on day 5 due to factors such as limited material space, inhibited cell proliferation and apoptosis. According to the results of MTT, the HAAM has good biocompatibility and can effectively support cell expansion.

### 3.2. Characterization of the Composite Scaffolds

**Figure 4:**
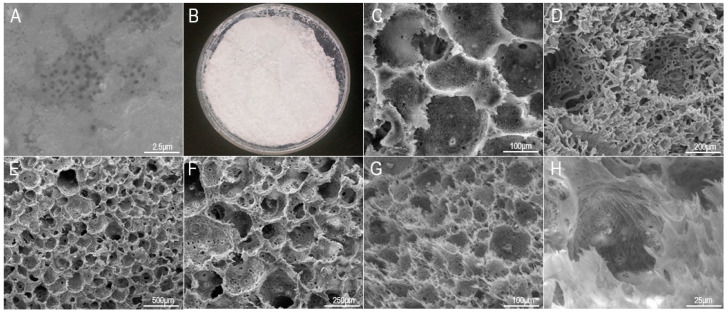
Characterization of HAAM and compound scaffolds. (**A**) HAAM under SEM; (**B**) Macroscopic morphology of HAAM after freeze drying; (**C**,**D**) 6%, 12% PLA scaffolds, (**E**,**F**) 6% PLA +3% nHAp scaffolds and (**G**,**H**) 6% PLA +3% nHAp +2% HAAM scaffold under SEM.

In the freeze-drying process, the acellular matrix does not undergo high-temperature treatment, and therefore can avoid the inactivation of proteins and retain its unique three-dimensional structure and mechanical properties [32], thus preserving the excellent properties of HAAM. The freeze-dried HAAM is white and smooth in texture (Figure 4B). With good rehydration ability, it can absorb water quickly and be restored to its elastic state before freeze-drying. Under SEM, it was observed that the HAAM stromal layer was exposed; it has a network structure and consists of one collagen fiber alternately stacked (Figure 4A). The emergence of the stromal layer can give full play to the advantages of HAAM and provide more sites for cell attachment. Cytokines in the stromal layer can also play a regulatory role in cell proliferation and differentiation.

Micropores of 150–200 μm are suitable for solid bone tissue growth, pores of 75–100 μm are suitable for non-mineralized bone tissue growth, and micropores of 10–75 μm are suitable for fibrous tissue penetration and cell crawling. As shown in Figure 4C, 6% of the PLA scaffold had a permeable pore structure with a pore size of about 200 μm, which provided sufficient space for the adhesion, proliferation and bone differentiation of osteoblasts and was suitable for the growth of solid bone tissue. It was found that there were micropore structures with a diameter of about 30 μm on the pore wall of the macropore, which could improve the mass transfer performance of the scaffold materials and facilitate the transfer of nutrients and metabolic waste [33]. In addition, studies have shown that the microporous structure on scaffolds can provide abundant attachment sites for cell adhesion and crawling [34]. These microporous structures can also provide a breakthrough for the penetration and growth of fibrous tissue so that the scaffold can be closely combined with new bone tissue and further induce the growth of new bone tissue. However, after the concentration of PLA was increased to 12%, as shown in Figure 4D, the pore wall of the scaffold was obviously thickened, which inevitably led to a decrease in porosity, and thus the space available for cell proliferation and differentiation was severely reduced.

The microstructure of scaffold materials did not change significantly after the addition of nHAp (Figure 4E,F). As can be seen from Figure 4G,H, the microporous structure still exists after the addition of HAAM, and a large number of extended fibrous edges can be observed at the edge of the pore wall, indicating that HAAM has been successfully doped into the scaffold. This uneven fibrous extension can further increase the attachment sites on the scaffold surface, and the superior water absorption of HAAM can also improve the ability of the scaffold to load nutrients, providing nutrient reserves for cell proliferation and differentiation.

The adsorption capacity for proteins plays an important role in adhesion, migration, proliferation and differentiation of seed cells [35]. When tissue-engineered implants come into contact with serum or body fluids, the material surface first comes into contact with water and inorganic salts. After that, the water layer on the material surface will directly affect the adsorption of proteins and other molecules in subsequent contact. Finally, cells will contact the scaffold surface covered by proteins and complete the adhesion [36]. Therefore, good protein adsorption capacity is an important prerequisite for tissue engineering scaffolds to attach cells and guide new bone generation.

The types of protein adsorption can be divided into non-specific adsorption and specific adsorption according to the driving force of adsorption. Non-specific adsorption refers to the adsorption of proteins by generating undifferentiated interactions with proteins, such as electrostatic force [37], van der Waals force, hydrogen bonding and hydrophilic interaction [38]. Specific adsorption refers to the selective and highly accurate adsorption of a specific protein, such as antibody–antigen binding [39]. The protein adsorption in this study was realized by the first kind of non-specific adsorption, so the determination of the adsorption capacity of the composite scaffold on bovine serum albumin (BSA) can better represent the adsorption capacity of the composite scaffold on a variety of proteins [40].

The 9 data corresponding to I, II and III in Table 3 are PLA content 4% (AI), 6% (AII), 8% (AIII), HAAM content 1% (BI), 2% (BII), 3% (BIII), nHAp content 2% (BI), 3% (BII), 4% (BIII), the respective sum of the adsorption capacity of scaffold protein per unit mass obtained from the one factor (a fixed material content) and three levels (other materials change contents) experiments. The data corresponding to K1, K2 and K3 are the average of the above data, respectively, and the data corresponding to factor A (PLA) in the table, I, II and III and K1, K2, K3, respectively reflect the influence of PLA content 4%, 6%, 8% levels on the adsorption capacity of scaffold protein per unit mass. Similarly, the data of I, II and III and K1, K2 and K3 corresponding to B(C) factor reflect the influence of HAAM content 1%, 2%, 3% (nHAp content 2%, 3%, 4%) levels on the adsorption capacity of scaffold protein per unit mass, respectively. According to the F value in Table 3, the influence order of all factors on the adsorption capacity of scaffold protein per unit mass is as follows: HAAM content > PLA content > nHAp content, that is, the main factor affecting the adsorption capacity of scaffold protein per unit mass is HAAM content, followed by PLA content; nHAp content has little effect on the adsorption capacity of scaffold protein per unit mass. According to the analysis, the optimal level combination of the composite scaffold preparation was a PLA concentration of 6%, a HAAM concentration of 2%, and a nHAp concentration of 3%. The scaffold prepared under these conditions had the maximum protein adsorption capacity per unit mass.

**Figure 5:**
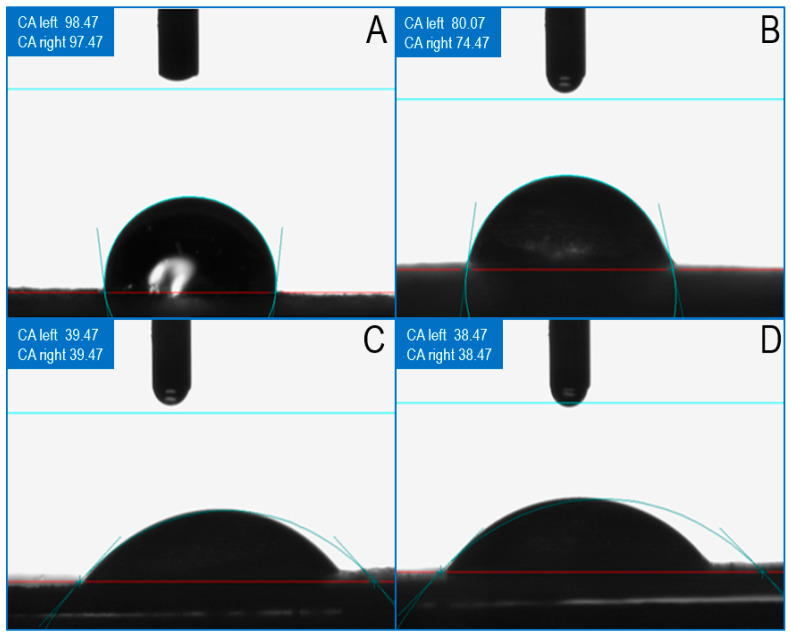
Contact angle with water of different scaffolds. (**A**) 6% PLA film; (**B**) 6% PLA +3% nHAp film; (**C**) 6% PLA +3% nHAp +2% HAAM film; (**D**) 6% PLA +3% nHAp +6% HAAM film.

Liquid infiltration on the scaffold surface is an important prerequisite for cell adhesion, so good hydrophilicity will be more conducive to cell adhesion. In addition, after implantation of tissue engineering scaffolds, they will directly contact with blood or tissue fluid [41] and allow nutrients to be transferred to seed cells through the pore structure of composite scaffolds or scaffolds themselves. Therefore, the water absorption performance of scaffolds is also an important performance index of tissue engineering scaffolds. As shown in Figure 5, from a macro perspective, the hydrophilicity of pure PLA material is at a minimum when the contact angle is greater than 90°. After adding nHAp, the contact angle decreases somewhat. However, when HAAM is added, water droplets are quickly absorbed by the material, and the contact angle also decreases rapidly with the absorption of water, indicating that the water absorption of HAAM promotes the infiltration of liquid into the material.

### 3.3. Cell proliferation and Viability Analysis of the Composite Scaffolds

**Figure 6:**
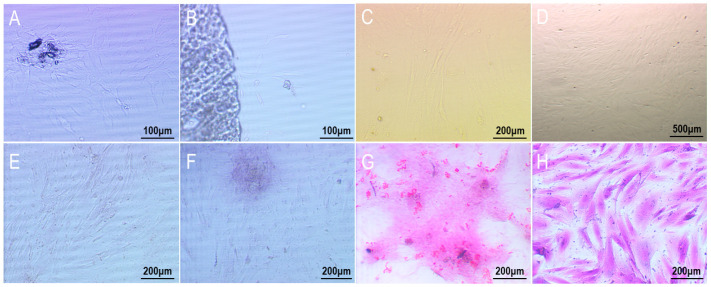
Staining observation and microscope photographs of osteoblasts’ primary culture and continuous culture. (**A**,**B**) Primary osteoblasts on the 2nd and 5th day, respectively; (**C**,**D**) P4 osteoblasts on the 2nd and 4th day, respectively; (**E**,**F**) ALP staining; (**G**) Alizarin red staining; (**H**) H&E staining.

As shown in Figure 6A,B, on day 2, some osteoblasts had crawled out of the bone fragments of the cranial cover and were in good adherent condition; on day 5, a large number of osteoblasts were observed around the bone fragments of the cranial cover, with polygonal cells, large nuclei and high purity, but not many cellular synapses and cellular networks had been formed. Figure 6C,D shows the morphology of osteoblasts in the fourth generation. On day 2, the cells had fully adhered to the wall and formed long cell synapses in the form of long spindles, and the cells close to each other had begun to contact each other. On day 4, the osteoblasts had spread all over the bottom of the culture bottle, but the cells were still in the form of long spindles, and the cells were highly connected at this time; a dense spiral network of cells was formed. 

Osteoblasts were stained with H&E to observe the cell morphology more directly. In addition, ALP staining and alizarin red staining were performed to characterize the osteogenic ability of osteoblasts. ALP is an exoenzyme secreted by osteoblasts, which plays an important role in the construction of tissue-engineered bone. First, it can hydrolyze phosphate esters into phosphoric acid for nHAp precipitation. In addition, it can hydrolyze pyrophosphate, thus eliminating its inhibition of calcium salt precipitation and promoting the formation of calcium nodules. Therefore, the amount of ALP can directly reflect the strength of osteoblast ability [42]. Calcium nodules are produced during the maturation of osteoblasts, and these nodules are an important basis for the occurrence of new bone. As an anthraquinone compound, alizarin red reacts with calcium nodules to produce red compounds, so that the growth of calcium nodules can be observed under the microscope.

As can be seen from Figure 6E, osteoblasts are in a vortex covering the bottom of the pore plate. The cells are polygonal and form long spindles, and the presence of nuclei dyed dark red can be observed. The cytoplasm of osteoblasts showed a gray-black chromogenic reaction, and large gray-black aggregates could also be observed (Figure 6F), which indicated that osteoblasts secreted more ALP. Figure 6G showed a large number of red nodules around osteoblasts, indicating that osteoblasts can still produce a large number of calcium nodules when cultured in vitro, and the distribution of these calcium nodules in flakes lays a good foundation for the occurrence of new bone. Therefore, osteoblasts extracted in this experiment have strong proliferation ability and can give full play to their osteoblastic ability, making them suitable for use as seed cells for bone tissue engineering.

**Figure 7:**
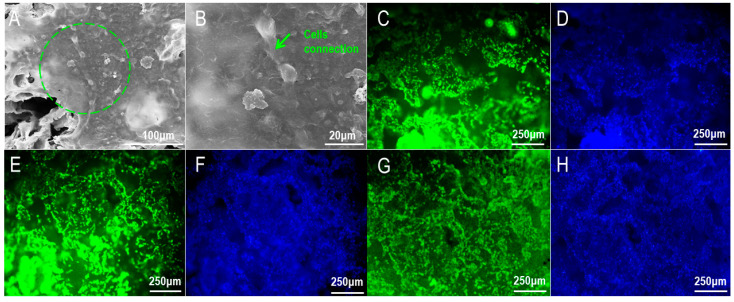
Viability and distribution of osteoblasts on the scaffold. (**A**,**B**) SEM of cell–scaffold compound (**A**: ×1000; **B**: ×3000, The circles represented the zoom position); Hochest and Calcein-AM stainings of (**C**,**D**) 6% PLA +3% nHAp +2% HAAM; (**E**,**F**): 6% PLA +3% nHAp; (**G**,**H**) 6% PLA.

The cell–scaffold complex was observed under SEM (Figure 7), cells were evenly distributed and fully spread on the surface of the scaffold, and cell synapses were fully fused with the scaffold. In addition, it can be seen from Figure 7B that synaptic connections have been established between cells, indicating that the cells proliferated well after adhesion to the scaffold, and there was a tendency for the cells to interconnect; this was conducive to the transmission of signaling factors between cells.

Compared with Figure 7D,F,H, it can be seen that the blue fluorescence on the 6% PLA scaffold is significantly less than that on the other two groups, indicating that the pure PLA scaffold is not ideal for the adhesion of osteoblasts. However, in 6% PLA +3% nHAp +2% HAAM and 6% PLA +3% nHAp scaffolds, osteoblasts were able to achieve a large amount of adhesion and distributed evenly, indicating that all components in the scaffold were uniformly dispersed and there was no large local agglomeration of a certain component. As can be seen from Figure 7C,E,G, large amounts of green fluorescence exist on the scaffolds of 6% PLA +3% nHAp +2% HAAM and 6% PLA +3% nHAp, indicating that osteoblasts can maintain good activity on the scaffolds of these two groups. However, in comparing Figure 7C,E, the fluorescent staining of osteoblasts on the 6% PLA +3% nHAp +2% HAAM scaffold was mostly spot-like, while on the 6% PLA +3% nHAp +2% HAAM scaffold, osteoblasts showed polygon and long spindle cell morphology, and the cells were more dense; the synaptic connections between cells were clearly visible (Figure 7E). These results indicated that the 6% PLA +3% nHAp +2% HAAM scaffold could support the adhesion and proliferation of osteoblasts in vitro.

**Figure 8:**
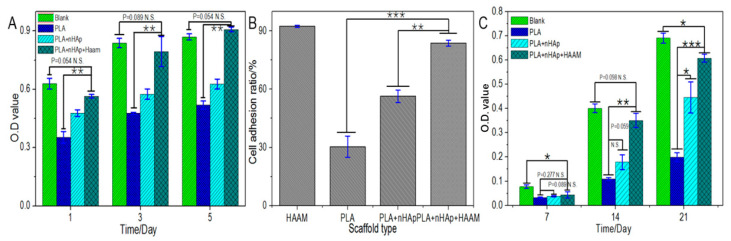
Osteoblasts’ proliferation (**A**) on different scaffolds (**B**), adhesion rate (**C**) and ALP quantitative testing. Data are indicated by mean ± SD. *: *p* < 0.05; **: *p* < 0.01; ***: *p* < 0.001.

Osteoblasts showed an increasing trend in the scaffolds and pore plates of all groups, and the number of cells reached the highest on day 5 (Figure 8A). Specifically, the number of cells on the 6% PLA +3% nHAp +2% HAAM scaffold was lower than that of the blank group (a blank well plate hole without scaffolds added) at day 1 and day 3. The absorbance rate reached 0.5549 and 0.6333 at day 1 and reached 0.7550 and 0.8123 at day 3, respectively, but the opposite result was seen at day 5, when the absorbance rate reached 0.9020 and 0.8534, respectively. The reason may be that in the first 3 days, the cells did not cover the bottom of the pore plate with sufficient growth space. However, contact inhibition occurred in the control group as the number of cells increased. Due to the high porosity of the scaffold, the number of cells on the 6% PLA +3% nHAp +2% HAAM scaffold exceeded that on the blank group on the 5th day. In addition, the number of cells on the composite scaffold with HAAM and nHAp was significantly higher than that on the pure PLA group in the first 5 days, indicating that the addition of HAAM and nHAp was conducive to the proliferation of osteoblasts.

Figure 8B shows that osteoblasts have the highest 4 h adhesion rate on HAAM, up to 92.33%, which again indicates that HAAM has superior cell adhesion ability. Among the three groups of scaffolds, pure PLA scaffolds had the lowest adhesion rate, only 30.34%. After the addition of nHAp, the cell adhesion rate of scaffolds increased to 56.34%, indicating that the addition of nHAp provided more abundant adhesion sites for osteoblasts. In the composite scaffolds with both HAAM and nHAp, the adhesion rate reached 83.67%. It was significantly higher than pure PLA scaffolds and PLA+nHAp scaffolds, and achieved a large number of osteoblast adhesion in a short time.

ALP is an enzyme protein secreted by osteoblasts. In bone tissue engineering, ALP is one of the most commonly used indicators to characterize the specific differentiation and secretion function of osteoblasts. Quantitative ALP detection was performed on osteoblasts planted on different scaffolds on the 7th, 14th and 21st day, respectively, as shown in Figure 8C. In general, the amount of ALP secreted by osteoblasts on scaffolds of each group increased with time. At day 7, there was no significant difference between the stent groups, and the ALP content was lower than that of the blank group (a blank well plate hole without scaffolds added) inoculated on the well plate. This is because the spatial structure of the scaffold is relatively complex. Osteoblasts implanted on the scaffold need to adhere to and crawl on the scaffold in the early stage, and experience a long plateau period, which leads to the delayed secretion of ALP. The content of ALP in the PLA+nHAp+HAAM group was significantly higher than that in the pure PLA group, and there was no significant difference between the PLA+NHAP+HAAM group and the blank group, indicating that osteoblasts inoculated on the scaffold could secrete a large amount of ALP after 14 days of culture. The addition of HAAM can significantly promote the secretion of ALP, but the addition of nHAp can increase the secretion of ALP; however, it has no significant effect compared with pure PLA. On the 21st day, the content of ALP in the PLA+nHAp+HAAM group was significantly higher than that in the pure PLA group, and nHAp also significantly promoted the secretion of ALP, indicating that both HAAM and nHAp could promote the secretion of ALP in osteoblasts to a great extent. These results indicated that osteoblasts maintained good differentiation and secretion ability on the PLA+nHAp+HAAM composite scaffold.

## 4. Conclusions

In this study, acellular amniotic membranes were used to prepare bone tissue engineering composite scaffolds with good bone induction and bone compatibility. Through comprehensive comparison, hypertonic saline +0.25% trypsin/EDTA solution was selected as the best method of four acellular amniotic membrane preparation methods. Our result showed that HAAM contained a large amount of high purity collagen and maintained a complete helical structure and functional groups. The cell viability assay of osteoblasts showed that the acellular amniotic membrane prepared in this study had excellent biocompatibility and could be used for the preparation of bone tissue engineering scaffolds. In conclusion, a PLA/nHAp/HAAM composite scaffold can greatly promote the proliferation, differentiation and protein expression of osteoblasts, and is an excellent material for the repair of bone defects in tissue engineering.

## Figures and Tables

**Table 1:** Orthogonal design.

Number	A	B	C
	PLA Concentration (W/V)	HAAM Concentration (W/V)	nHAp Concentration (W/V)
1	8%	3%	2%
2	4%	2%	4%
3	8%	1%	4%
4	4%	3%	3%
5	6%	3%	4%
6	8%	2%	3%
7	6%	2%	2%
8	6%	1%	3%
9	4%	1%	2%

**Table 2:** Characteristic absorption peaks in the infrared spectrum of HAAM.

Absorption Band	Characteristic Group	Frequency (cm^−1^)	Type of Vibration
Amide A	N-H	3388	υs
Amide I	C=O	1668	υs
Amide II	N-H C-H	1546 2948	β υs
	-CH_3_	1454	β
	C-N-C\C-O	1137	υ
Amide IV	C-N\N-H	1072	υ

**Table 3:** Orthogonal test results.

Number	A	B	C	Protein Adsorption Capacity (mg/g)
	PLA Concentration (W/V)	HAAM Concentration (W/V)	nHAp Concentration (W/V)	
1	8%	3%	2%	25.44
2	4%	2%	4%	31.95
3	8%	1%	4%	29.57
4	4%	3%	3%	28.98
5	6%	3%	4%	27.70
6	8%	2%	3%	32.37
7	6%	2%	2%	32.17
8	6%	1%	3%	30.12
9	4%	1%	2%	28.93
I	89.86	88.62	86.54	-
II	89.99	96.49	91.47	-
III	87.38	82.12	89.22	-
K_1_	29.96	29.54	28.85	-
K_2_	29.99	32.17	30.49	-
K_3_	29.13	27.37	29.74	-
F	1.723	22.181	1.361	-
Optimal level	AII (PLA 6%)	BII (HAAM 2%)	CII (nHAp 3%)	-

## Data Availability

Not applicable.
